# A Quantitative Data-Driven Analysis Framework for Resting-State Functional Magnetic Resonance Imaging: A Study of the Impact of Adult Age

**DOI:** 10.3389/fnins.2021.768418

**Published:** 2021-10-20

**Authors:** Xia Li, Håkan Fischer, Amirhossein Manzouri, Kristoffer N. T. Månsson, Tie-Qiang Li

**Affiliations:** ^1^Institute of Informatics Engineering, China Jiliang University, Hangzhou, China; ^2^Department of Psychology, Stockholm University, Stockholm, Sweden; ^3^Stockholm University Brain Imaging Centre, Stockholm, Sweden; ^4^Centre of Psychiatry Research, Department of Clinical Neuroscience, Karolinska Institutet, Stockholm, Sweden; ^5^Department of Clinical Science, Intervention, and Technology, Karolinska Institute, Solna, Sweden; ^6^Department of Medical Radiation and Nuclear Medicine, Karolinska University Hospital, Solna, Sweden

**Keywords:** quantitative data-driven analysis (QDA), resting-state functional magnetic resonance imaging (R-fMRI), resting-state functional connectivity (RFC), connectivity strength index (CSI), connectivity density index (CDI), adult age

## Abstract

The objective of this study is to introduce a new quantitative data-driven analysis (QDA) framework for the analysis of resting-state fMRI (R-fMRI) and use it to investigate the effect of adult age on resting-state functional connectivity (RFC). Whole-brain R-fMRI measurements were conducted on a 3T clinical MRI scanner in 227 healthy adult volunteers (*N* = 227, aged 18–76 years old, male/female = 99/128). With the proposed QDA framework we derived two types of voxel-wise RFC metrics: the connectivity strength index and connectivity density index utilizing the convolutions of the cross-correlation histogram with different kernels. Furthermore, we assessed the negative and positive portions of these metrics separately. With the QDA framework we found age-related declines of RFC metrics in the superior and middle frontal gyri, posterior cingulate cortex (PCC), right insula and inferior parietal lobule of the default mode network (DMN), which resembles previously reported results using other types of RFC data processing methods. Importantly, our new findings complement previously undocumented results in the following aspects: (1) the PCC and right insula are anti-correlated and tend to manifest simultaneously declines of both the negative and positive connectivity strength with subjects’ age; (2) separate assessment of the negative and positive RFC metrics provides enhanced sensitivity to the aging effect; and (3) the sensorimotor network depicts enhanced negative connectivity strength with the adult age. The proposed QDA framework can produce threshold-free and voxel-wise RFC metrics from R-fMRI data. The detected adult age effect is largely consistent with previously reported studies using different R-fMRI analysis approaches. Moreover, the separate assessment of the negative and positive contributions to the RFC metrics can enhance the RFC sensitivity and clarify some of the mixed results in the literature regarding to the DMN and sensorimotor network involvement in adult aging.

## Introduction

Among the different analysis approaches for resting-state fMRI (R-fMRI) data, the anatomic region-of-interest (ROI)-based, and data-driven independent component analysis (ICA) methods are probably the most commonly used (Tyszka et al., 2014). Resting-state functional connectivity (RFC) results from the ROI-based and ICA derived methods are generally similar but conceptually different. The quantitative relationship between ROI-based and ICA derived measures of RFC has been investigated with computer simulation and experiment approaches (Joel et al., 2011; Rosazza et al., 2012). In theory, the ROI-based RFC measures can be shown to be the sum of the ICA derived RFC both for the within and between networks (Joel et al., 2011; Rosazza et al., 2012).

With ROI-based analysis the brain is first parcellated into pre-defined anatomical regions, the mean time course for each ROI is then determined. By calculating the temporal correlations in a pairwise fashion between the defined ROIs, for each R-fMRI dataset a correlation coefficient matrix of the ROIs can be obtained for further statistical assessment. Therefore, specific connectivity between specific regions is explicitly tested in a model-driven framework by using the average time courses of the selected ROIs as a temporal model. Since the RFC patterns do not necessarily coincide precisely with the atlas-based ROI definition, all voxels within predefined ROIs are not necessarily a part of the network-of-interest and functionally connected. This can potentially affect the accuracy and sensitivity of the ROI-based analysis (Song et al., 2016). On the other hand, ICA can reveal dynamics and spatially distributed brain networks in a data-driven fashion without the need of a temporal model. Beside motor and sensory networks, ICA studies have identified the brain networks involved in attentional control (Lawrence et al., 2003), including the task-dependent (Vossel et al., 2014) dorsal and ventral lateral attention networks (DAN and VAN), the task-independent (Raichle et al., 2001; Buckner et al., 2008) default mode network (DMN), and the salience network (SN), which was postulated to be the switching control network for the up-regulation of attention networks and the downregulation of the DMN (Menon and Uddin, 2010). The dynamic interactions between the DAN, VAN, DMN, and SN networks are believed to be the key for understanding the function and dysfunction efficient attention allocation for task performance.

Despite the growing consensus regarding the ICA-derived intrinsic RFC networks in the healthy brain with stable spatial components reproduced across studies (Damoiseaux et al., 2006; Smith et al., 2009; Allen et al., 2011), the precise number of independent components (NIC), as a prerequisite input parameter for ICA, is not known *a priori.* NIC can substantially influence the ICA outcomes (Wang and Li, 2015). Moreover, there is lack of gold standard for the selection of meaningful components to exclude non-interesting noise resources, such as ventricular, vascular, susceptibility, or motion-related artifacts (Wang and Li, 2013).

In this study we refined further of our quantitative data-driven analysis (QDA) framework based on the time course of individual voxel inside the brain. The QDA approach is data-driven as ICA and can generate two types of quantitative RFC metrics for each voxel inside the brain without the need for specifying a particular threshold, model or mode. Since it uses the time course of each voxel within the brain as the reference seed in turn to compute voxel-wise whole-brain correlational coefficient matrix, the size of the correlation matrix is equal to the number of voxels inside the brain. It is typical *N* > 10^4^ for whole-brain R-fMRI datasets with 4 mm voxel size. To facilitate further statistical assessment of the whole-brain correlation matrix, we derive two types of voxel-wise RFC metrics from the correlation matrix, namely the connectivity strength index (CSI) and connectivity density index (CDI). CSI and CDI provide general connectivity metrics of strength and density for the local voxel with the rest of brain, respectively. These metrics can be used for straightforward statistical comparison to assess differences between groups and longitudinal changes of individuals. This is a basic requirement for radiological diagnosis in clinical practice.

Several voxel-based RFC metrics have been proposed in the literature. Among other things, the regional homogeneity (Zang et al., 2004; Meier et al., 2017; Reynolds et al., 2017), measures of low frequency oscillation including the amplitude of low frequency fluctuations (ALFF) and the fractional ALFF (Yang et al., 2007; Zang et al., 2007; Zou et al., 2008; Sun et al., 2016; Zhang X.D. et al., 2016; Pan et al., 2017), measurements of complexity, such as the Hurst exponent (Hayasaka and Laurienti, 2010; He, 2011; Ciuciu et al., 2014), and brain entropy (de Araujo et al., 2003; Zhao et al., 2010; Jia et al., 2017; Viol et al., 2017) have been used for studying the RFC in normal and diseased brains. These methods have yielded interesting results. However, there remains still some methodological issues to be addressed, such as the arbitrariness in the selection of cut-off frequency (Yang et al., 2007; Zang et al., 2007; Zou et al., 2008; Sun et al., 2016; Zhang X.D. et al., 2016; Pan et al., 2017), loss of information (Hayasaka and Laurienti, 2010; He, 2011; Ciuciu et al., 2014), and computation difficulty (de Araujo et al., 2003; Zhao et al., 2010; Jia et al., 2017; Viol et al., 2017). These technical difficulties may have contributed to the inconsistent findings in the published literature. Moreover, the different RFC metrics portray different aspects of R-fMRI signal and may have different sensitivities to the physiological activities and pathological abnormality (Golestani et al., 2017; Reynolds et al., 2017).

Both ICA and ROI-based approaches have previously been applied to study age-related changes in RFC (Bluhm et al., 2008; Biswal et al., 2010; Weissman-Fogel et al., 2010; Zuo et al., 2010; Dennis and Thompson, 2014; Alarcon et al., 2015; Zhang C. et al., 2016). Numerous studies have confirmed that reduced RFC in healthy aging in the DMN is correlated with cognitive deficit (Damoiseaux et al., 2008; Biswal et al., 2010; Campbell et al., 2013; Ferreira and Busatto, 2013; Scheinost et al., 2015). There is accumulating evidence to support the notion that elderly adults typically have reduced RFC across most parts of the DMN, particularly in the dorsal medial prefrontal cortex (mPFC) and the ventral and posterior cingulate cortex (PCC; Campbell et al., 2013; Scheinost et al., 2015). However, in the reported literature there is also considerable variability concerning age-related RFC differences in the limbic and other DMN subsystems. For example, some studies have found age-related RFC reduction in the hippocampal (Damoiseaux et al., 2008; Campbell et al., 2013; Scheinost et al., 2015) and subcortical regions (Ystad et al., 2010), whereas others reported either no significant decline or elevated RFC in some of the specific hippocampal (Pasquini et al., 2015) and DMN regions (Salami et al., 2014; Damoiseaux et al., 2016). The discrepancies in the reported findings among the different R-fMRI studies may reflect not only variability in the sample characteristics, but also diversity in the data processing methods for deriving the different RFC metrics for connectivity of specific pathways.

The main objective of this study is to develop a QDA framework to analyze R-fMRI data and derive quantitative, model-free, and threshold-free RFC metrics, which are optimally sensitive to physiological and pathological changes in the central nervous systems. We used the proposed metrics to assess if and how adult age in healthy subjects influences these RFC metrics.

## Experimental and Methods

### Participants

A total of 227 volunteers (aged 18–76 years, male/female = 99/128) completed the study and were recruited into the study through the local media advertisement in the Stockholm region. All participants were right-handed, and native Swedish speakers with normal or corrected-to-normal vision. They all reported being free of a history of neurological, psychiatric, and cardiovascular diseases. None of the participants reported any use of psychotropic drugs. Each subject signed informed consent before completing the magnetic resonance imaging (MRI) examination protocol. They were financially compensated for their participation. The regional ethics committee approved the study protocol 2014/1982-31/1, which was conducted in line with the declaration of Helsinki.

### Magnetic Resonance Imaging Data Acquisition Protocol

The MRI data acquisition was conducted on a whole-body 3T clinical MRI scanner (Magnetom Trio, Siemens Medical Solutions, Erlangen, Germany) equipped with a 32-channel phased-array receiving head coil. All data was acquired at Karolinska University Hospital, Huddinge, Stockholm, between noon and 5:00 PM. The MRI data acquisition protocol included the following scanning sessions: (1) 3-plane localizer; (2) Conventional clinical MRI scans including 3D T1-weighted MPRAGE, T2 and FLAIR scans; and (3) A session of 375 s long R-fMRI measurements. The main acquisition parameters for the R-fMRI data included the following: TE/TR 35/2,500 ms, flip angle = 90°, 34 slices of 3.5 mm thick, FOV = 225 mm, matrix size = 76 × 76, data acquisition acceleration with GRAPPA parallel imaging method (iPAT = 2), and 150 dynamic timeframes. The T1-weighted MPRAGE images used for co-registration with functional images were acquired with the following parameters: TR = 1,900 ms, TE = 2.52 ms, FA = 9 degrees, FOV = 256, voxel size 1 mm × 1 mm × 1 mm. The acquisition parameters for the FLAIR image were the following: TE/TR = 89/9,000 ms, flip angle = 130°; inversion time (TI) = 2,500 ms, slice thickness = 4.0 mm, and FOV = 199 × 220 mm. An experienced radiologist inspected both the FLAIR and T1-weighted images for potential signs of neuropathology.

We used foam patting to fix each subject’s head carefully in the head coil to reduce involuntary head motions. During the R-fMRI data acquisition the participants were instructed to focus their sight on a white cross in black background projected on a screen installed in front of their eyes. The subjects were also instructed to not think about anything particular during the R-fMRI session.

### Resting-State Functional Magnetic Resonance Imaging Data Pre-processing

The R-fMRI datasets underwent a preprocessing procedure, which has been described elsewhere in details (Li et al., 2021) and was performed with AFNI (Version Debian-16.2.07∼dfsg.1-3∼nd14.04+1, http://afni.nimh.nih.gov/afni) and FSL^1^ programs with a bash wrapper shell (Wang and Li, 2013, 2015). After temporal de-spiking, six-parameter rigid body image registration was performed for motion correction. The average volume for each motion-corrected time series was used to generate a brain mask to minimize the inclusion of the extra-cerebral tissues. Spatial normalization to the standard MNI template was performed using a 12-parameter affine transformation and a mutual-information cost function. During the affine transformation the imaging data were also re-sampled to isotropic resolution using a Gaussian kernel with 4 mm full width at half maximum (FWHM). The co-registered average image volume for the cohort has 28,146 non-zero voxels inside the brain and was used to generate the average brain mask for the preprocessed whole-brain R-fMRI data with 4 mm spatial resolution. Nuisance signal removal was performed by voxel-wise regression using 14 regressors based on the motion correction parameters, average signal of the ventricles and their 1st order derivatives. After baseline trend removal up to the third order polynomial, effective band-pass filtering was performed using low-pass filtering at 0.08 Hz. Local Gaussian smoothing up to FWHM = 4 mm was performed using an eroded gray matter mask (Wang and Li, 2015).

**FIGURE 1:**
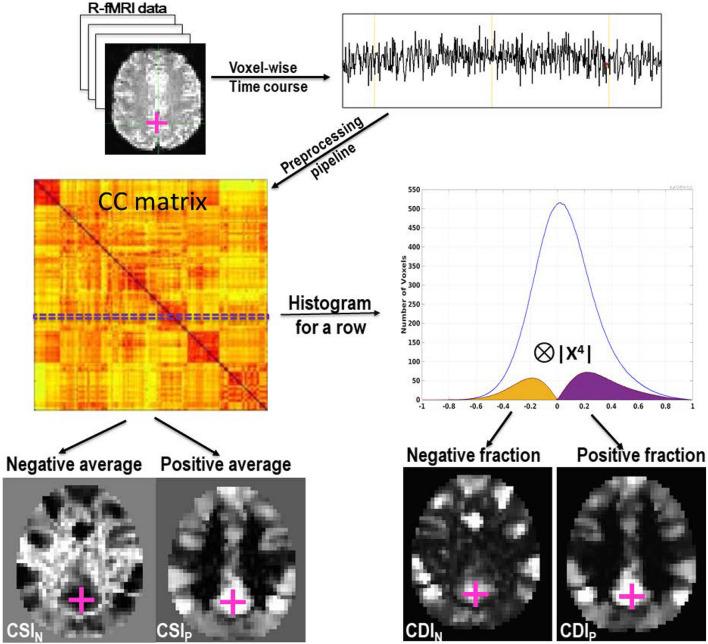
A schematic overview to illustrate the QDA framework. With QDA the time course of each voxel is used in turn to compute the whole-brain CC matrix. For each row of the CC matrix, we compute a CC histogram with 200 evenly binned intervals within [−1, 1]. The histogram shown in the graph is the cohort’s average CC histogram for a voxel within the PCC as marked with the cross. Two types of RFC images are derived from the CC matrix: (1) CSI_P_ and CSI_N_ whose voxel values are the averages of the positives and negatives in each row of the CC matrix, respectively. (2) CDI_P_ and CDI_N_ whose voxel values are the positive and negative parts of the convolution between the CC histogram and the kernel, respectively.

Pearson’s correlation coefficients (CC) were computed between the time courses of all pairs of voxels inside the brain, leading to a whole-brain functional connectivity matrix for each subject. This computation was performed for all voxels located within the brain mask, which was generated by overlapping the registered brains of all participants. This brain mask contained 28,146 voxels and each voxel inside the brain was used as the seed voxel in turn. Therefore, the size of the CC matrix size is 28,146 × 28,146. Each row or column of the CC matrix corresponds to the CC image volume for the seed voxel with the rest of the brain. That is the connectivity map for the seed voxel. As schematically illustrated in Figure 1, based on the CC histogram for each row of the matrix we derived the following two types of threshold-free voxel-wise RFC metrics: the CSI and CDI. As we are interested in systematically investigating all relevant synchronized activities in the whole brain, we quantify the negative and positive portions of the CC histogram separately to avoid information cancelation, sensitivity reduction, and statistical interference. From here on, the subscripts “N” and “P” are used to indicate the negative and positive portions of the RFC metrics, respectively. The metrics without subscripts refer to the mixed measures without distinction of the negative and position correlations.

As shown in Figure 1, the voxel value for the CSI_P_ and CSI_N_ are defined as the averages of the positives and negatives in each row of the CC matrix, respectively. That is


(1) $${\text{CSI}}_{\text{P}}=\left(\sum_{\mathrm{CC}>0}{\text{CC}}_{\text{row}}\right)/n\,p$$

(2) $${\text{CSI}}_{\text{N}}=\left(\sum_{\mathrm{CC}<0}{\text{CC}}_{\text{row}}\right)/n\,n$$

Where CC_row_ refers to a row in the CC matrix. *np* and *nn* refer to the number of positive and negative correlation coefficients in a row of the CC matrix, respectively. The voxel values for CDI are defined as the convolution between the CC histogram and a kernel function. That is


(3) $$\text{CDI}=\text{Hist}\,\left({\text{CC}}_{\text{row}}\right)⊗\text{kernel}$$

The CDI_P_ and CDI_N_ correspond to the positive and negative portions of the convolution defined in Eq. (3), respectively. To facilitate statistical comparison it is useful to transform the raw RFC metrics into standard *Z*-score using the following formula:


(4) Z=(RFC-u)/σ

**FIGURE 2:**
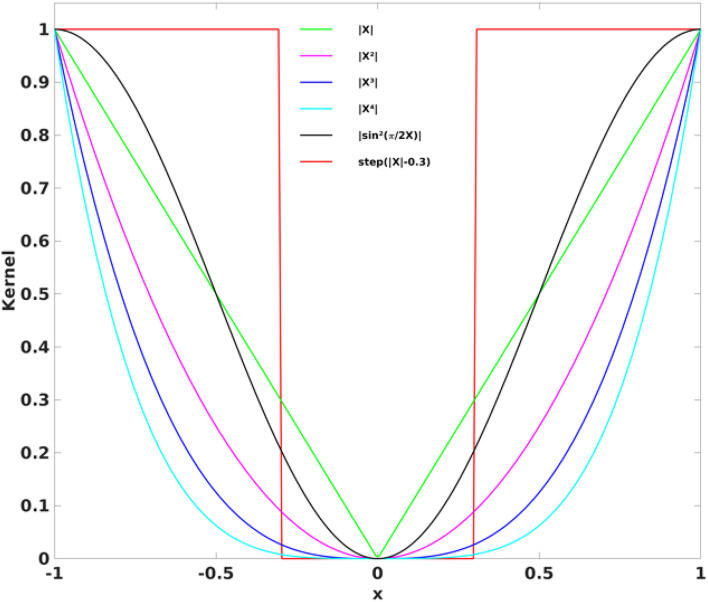
The six different kernel functions investigated in the study to derive the CDI_P_ and CDI_N_ metrics. The widely used threshold method can be considered as the case for the square-well kernel function (k_6_).

Where μ and σ are the mean and standard deviation of the corresponding RFC metrics, respectively. For optimization of the CDI sensitivity, we investigated 6 different kernel functions including


(5) $$k_{i=1,2,\ldots ,4}=\left|{\text{x}}^{i}\right|,$$

(6) $$k_{5}=\left|{\text{sin}}^{2}\,\left(\pi \,/\,2\,\text{x}\right)\right|,$$

(7) $$k_{6}=\text{step}\,\left(\left|\text{x}\right|-0.3\right),$$

where x⊂ [−1,1] corresponds to the interval of the correlation coefficients. The kernels are also graphically depicted in Figure 2. The kernel should weight the higher correlation coefficients more than the lower ones. The widely used threshold approach can be considered as the case of the square-well kernel function *k*_6_. For illustration, an arbitrary threshold of 0.3 was used here. The CSI metrics can also be considered as a special case of CDI corresponding to a kernel of the sign function.

### Statistical Analyses

To investigate if and how the RFC metrics are influenced by heathy aging for the studied cohort we performed voxel-wise linear regression analyses of the CSI and CDI metrics versus the subject’s age, while gender was treated as a covariate by using the AFNI program 3dRegAna to extract the regression parameter β and linear coefficient *r*. The statistical significance was assessed by using a two-step approach. Firstly, we imposed a voxel-wise threshold *p* < 0.001 (uncorrected corresponding *t*-score ≥ 3.34) to form the initial cluster candidates. Secondly, we performed permutation simulations without assuming a particular form of probability distribution for the voxel values in the statistic images to identify the brain ROI out of the initially detected clusters at family-wise error rate *p* ≤ 0.05. Using the detected ROIs as masks, the mean values of the RFC metrics for each ROI were evaluated and plotted against the subjects’ age. Besides linear regression analysis with age, we performed also verification using two-sample *t*-test between the young and elderly subgroups. For this, we selected all subjects aged 18–30 years as the young subgroup (*n* = 124, males/females = 51/73), and all subjects aged 64–76 years as the elderly subgroup (*n* = 76, males/females = 35/41). To keep sufficient age gap between the young and elderly subgroups the remaining 27 subjects in the age range of 31–63 years old were excluded from the *t*-test. In the selection of subgroups we attempted to minimize the number of excluded subjects with intermediate ages, maximize the age gap between the subgroups, and keep similar number of subjects and age ranges. It should be emphasized that all 227 subjects were included in the regression analysis.

## Results

### The Quantitative Data-Driven Analysis Framework

**FIGURE 3:**
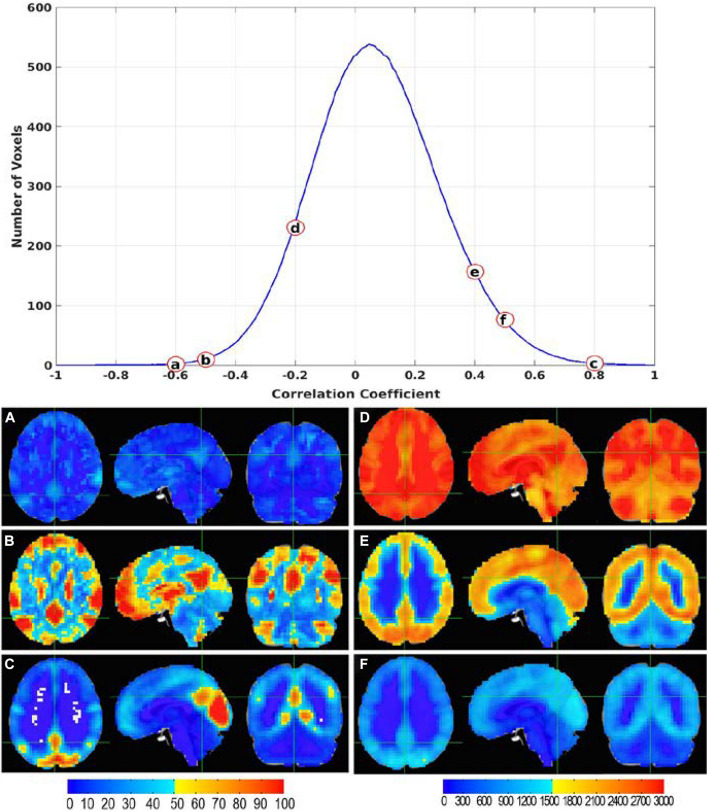
The average CC histogram of the cohort for a seed voxel in the posterior cingulate cortex (PCC) as indicated by the green cross. Selecting different threshold values along the histogram allows us to examine the functional connection networks of different strengths **(A–F)** associated with the seed voxel in the PCC.

The CC histogram for each seed voxel in the brain is dependent on its location in the brain (see Supplementary Materials). Figure 3 shows the average CC histogram of the cohort for a seed voxel in the PCC as illustrated by the cross in Figure 1. The histogram is somewhat asymmetric and shifted toward the positive side. This is quite typical at least for voxels within gray matter. Selecting different threshold values along the histogram allows us to examine the RFC networks of different connection strengths associated with the selected seed voxel. As shown in Figure 3, at high negative threshold (Figures 3A,B) we observe the DMN. At low negative threshold, we observe its association with cerebral spinal fluid (CSF) space and white matter (Figure 3D). At moderately high positive threshold, the PCC voxel is not only a part of the DMN, but also connected to most of the cortical gray matter (Figures 3E,F). At high positive threshold, the PCC voxel is associated with the posterior portion of the DMN and the visual cortex (Figure 3C). The visual cortex looks relatively bright at high threshold indicating a relatively high number of voxels are associated with the visual network or voxels within the visual cortex are associated with each other at high threshold criterion. The idea of the QDA framework is to avoid the arbitrary threshold and optimize the contribution of meaningful informatics to the quantitative RFC metrics.

**FIGURE 4:**
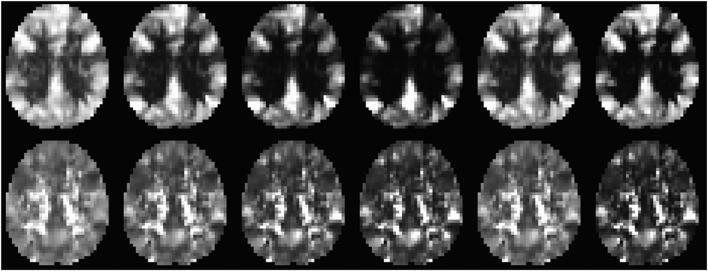
An axial slice of the CDI_P_ (upper row) and CDI_N_ (lower row) metrics derived from a typical R-fMRI dataset (a male subject of 36 year’s age). The images from left to right depict the results for the following 6 kernel functions | x|, | x^2^|, | x^3^|, | x^4^|, sin^2^(π/2x), and step(| x| −0.3), respectively.

Figure 4 shows an axial slice of CDI_P_ and CDI_N_ images for a typical R-fMRI dataset (from a 36 year old male subject). Multiple brain regions depict high CDI_P_ including the bilateral mPFC, superior and middle temporal gyri (MTG), inferior and superior parietal lobule, precuneus and PCC. These regions have been described as RFC hubs implying their important role in neural signaling and communication across the brain (Buckner et al., 2009; Tomasi and Volkow, 2011). On the other hand, the PCC, insula cortex. White matter and CSF regions have high CDI_N_ metric. The contrast and intensity variations across each row in Figure 4 demonstrate that selection of the kernel function can optimize the contrast and signal-to-noise ratio of the CDI metrics.

### Resting-State Functional Connectivity Changes Associated With the Adult Age

**FIGURE 5:**
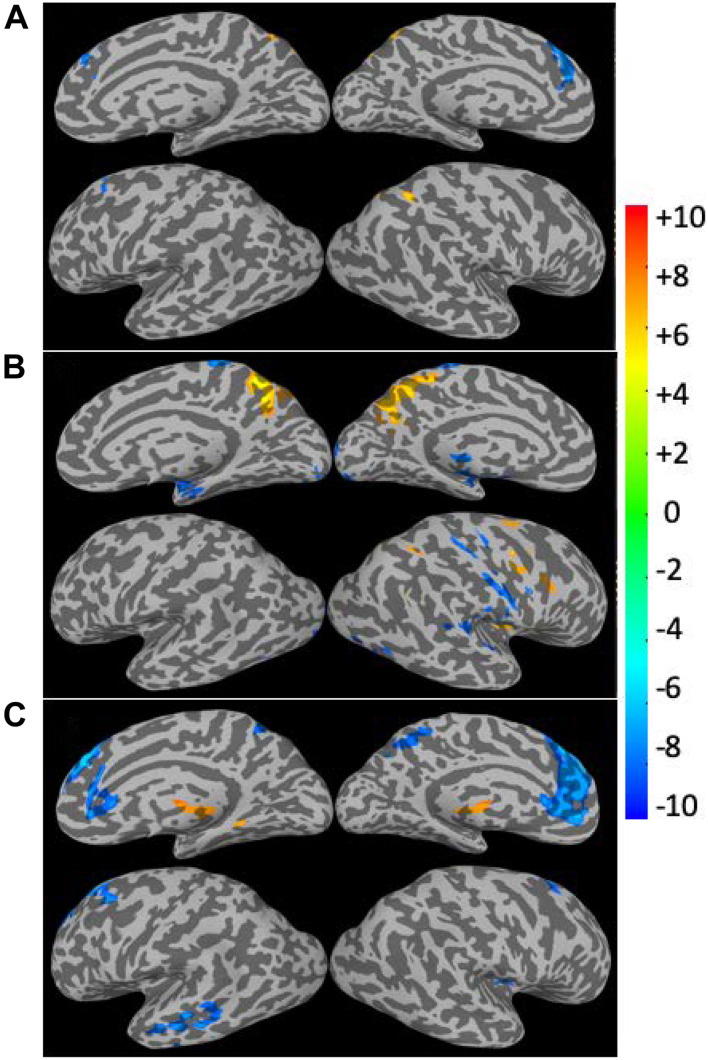
Brain regions with significant correlation (*p* < 0.05, corrected) between the connectivity strength metrics and the subject’s age. The results for the CSI **(A)**, CSI_N_ **(B)**, and CSI_P_ **(C)** are depicted separately. The Color bar shows the *t*-score level.

**FIGURE 6:**
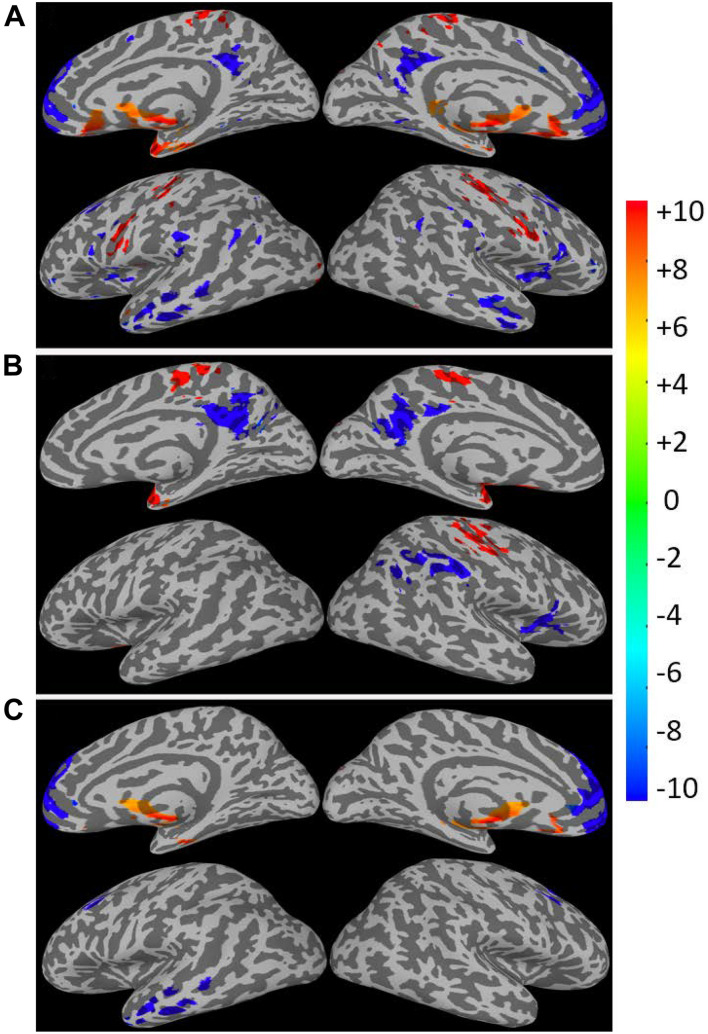
Brain regions with significant correlation (*p* < 0.05, corrected) between the connectivity strength metrics and the subject’s age. The results for the CDI **(A)**, CDI_N_ **(B)**, and CDI_P_ **(C)** are depicted separately. The Color bar shows the *t*-score level.

The linear regression results for the CSI, CSI_N_, and CSI_P_ data versus subjects’ age are summarized in Figure 5 and Table 1. The corresponding results for the CDI, CDI_N_, and CDI_P_ are shown in Figure 6 and Table 2. The CSI metric without separation of the negative and positive correlations shows decline of the functional connectivity strength with age in the superior and middle prefrontal gyrus (MFG) and increase of connectivity strength in the precuneus and right inferior parietal lobule (r-IPL). The more specifically defined CSI_N_ and CSI_P_ metrics are more sensitive to the adult age effect and the detected brain volumes with significant aging effect are nearly tripled compared with that for the CSI metric. With CSI_N_ and CSI_P_ we also observe a more intricate pattern of change with the adult age, which are summarized as follows:

**TABLE 1:** The brain regions where the connectivity strength metrics are significantly (*p* < 0.05) correlated with the subjects’ ages.

RFC	Voxel	*X*_cm_	*Y*_cm_	*Z*_cm_	β (10^3^)	*r*	*p*	Annotation
CSI	239	+ 2.1	+ 63.0	+ 47.9	9.50	0.459	<0.01	Precuneus
	152	+ 3.4	–47.3	+ 30.1	–9.72	–0.389	<0.01	Superior and MFG
	62	–38.5	+ 55.3	–35.5	9.57	0.354	<0.01	R-IPL
CSI_N_	237	+ 0.0	+ 53.1	+ 27.3	12.29	0.413	<0.01	PCC
	171	–42.6	–10.4	+ 11.0	12.36	0.411	<0.01	R-insula cortex
	161	–2.3	+ 28.2	+ 59.4	–11.05	–0.371	<0.01	Paracentral lobule
	153	–49.1	+ 47.6	+ 41.2	11.62	0.441	<0.01	R-IPL
	133	–39.8	+ 25.7	+ 55.5	–10.99	–0.325	<0.01	R-postcentral gyrus
	75	–27.3	–3.8	–34.0	–9.99	–0.450	<0.01	R-PHC
	67	–56.8	+ 14.6	+ 5.9	–9.28	–0.400	<0.01	R-STG
	58	+ 18.4	+ 0.4	–18.9	–9.36	–0.441	<0.01	L-PHC
	56	+ 42.0	+ 25.9	+ 57.1	–10.38	–0.309	<0.01	L-postcentral gyrus
	713	+ 2.0	–45.4	+ 23.6	–10.37	–0.487	<0.01	Superior and MFG
CSI_P_	157	–1.1	+ 14.7	–17.8	7.96	0.506	<0.01	Putamen
	110	+ 55.7	+ 13.1	–19.5	–9.49	–0.433	<0.01	L-MTG
	75	+ 2.7	+ 48.5	+ 31.1	–9.01	–0.336	<0.01	PCC
	53	–40.0	–8.7	+ 0.8	–8.48	–0.361	<0.01	R-insula cortex
	52	+ 45.1	–10.5	–8.9	–8.36	–0.376	<0.01	L-insula cortex
CSI_N_ CSI_P_	70	+ 2.8	+ 49.0	+ 31.0	14.15	0.374	<0.01	PCC (CSI_N_)
					13.37	0.362	<0.01	PCC (CSI_P_)
	34	–40.8	–10.0	+ 0.0	–9.04	–0.334	<0.01	R-Insula cortex (CSI_N_)
					–8.63	–0.336	<0.01	R-Insula cortex (CSI_P_)

*The volume, center of mass coordinates in MNI space, regression parameter (β), linear correlation coefficient (*r*), statistical significance (*p*), and anatomic annotations are specified. The default is bilateral, while R and L- indicate the right and left hemisphere of the brain, respectively. CSINP indicates the overlapping results between CSIN and CSIP.*

**TABLE 2:** The brain regions where the connectivity strength metrics are significantly (*p* < 0.05) correlated with the subjects’ ages.

RFC	Voxel	*X*_cm_	*Y*_cm_	*Z*_cm_	β (10^3^)	*r*	*p*(10^–3^)	Annotation
CDI	736	+ 0.7	–45.4	+ 24.2	6.511	0.603	<0.01	Superior amd MFG
	663	–6.0	+ 5.6	–19.3	–13.43	–0.469	<0.01	Caudate/putamen
	100	+ 0.7	+ 48.4	+ 30.8	–14.14	–0.325	<0.01	Precuneus
	99	+ 56.3	+ 16.8	–16.2	–11.04	–0.412	<0.01	L-ITG
	86	–45.9	–10.2	+ 6.6	–10.81	–0.387	<0.01	R-insular cortex
	63	–2.7	+ 33.0	+ 66.3	11.48	0.306	<0.01	r-postcentral gyrus
	49	–58.3	+ 15.3	–16.8	–10.83	–0.408	<0.01	R-ITG
CDI_N_	243	–0.0	+ 52.4	+ 29.9	–14.78	–0.381	<0.01	PCC
	124	–2.3	+ 28.5	+ 58.3	11.49	0.354	<0.01	Primary motor cortex
	98	–48.4	+ 47.3	+ 42.1	–14.41	–0.426	<0.01	R-IPL
	96	–40.1	+ 25.2	+ 55.8	12.09	0.327	<0.01	R-postcentral gyrus
	85	+ 20.5	–2.2	–20.7	10.52	0.423	<0.01	L-piriform cortex
	83	–44.3	–13.1	+ 3.1	–16.74	–0.375	<0.01	R-insular cortex
	56	–27.3	–6.6	–31.4	10.12	0.403	<0.01	R-fusiform gyrus
	54	+ 40.8	+ 25.1	+ 57.6	10.00	0.302	<0.01	L-postcentral gyrus
	39	+ 38.1	–12.3	+ 4.2	–13.85	–0.381	<0.01	L-insular cortex
	37	+ 57.0	+ 9.2	+ 30.8	10.72	0.315	<0.01	L-postcentral gyrus
	36	+ 47.6	+ 19.1	+ 6.2	8.68	0.376	<0.01	L-STG
	36	–7.6	–14.3	+ 40.1	–11.73	–0.349	<0.01	ACC
	31	–47.2	+ 21.0	+ 8.2	8.32	0.382	<0.01	R-STG
	804	+ 2.0	–45.3	+ 24.8	–13.14	–0.476	<0.01	Superior and MFG
	485	+ 1.1	+ 8.*r*05	–5.0	5.69	0.577	<0.01	Caudate/putamen
CDI_P_	92	+ 55.9	+ 14.2	–18.2	–10.23	–0.400	<0.01	L-ITG
	50	–30.2	–9.1	–33.3	7.53	0.388	<0.01	R-ITG
	30	+ 0.6	+ 46.2	+ 33.2	–12.87	–0.303	<0.01	PCC
CDI_N_CDI	23	–28.7	–8.5	–32.1	10.81	0.372	<0.01	R-parahippocampal (CDI_N_)
					7.186	0.376	<0.01	R-parahippocampal (CDI_P_)
	23	+ 0.2	+ 47.2	+ 33.3	–17.27	–0.330	<0.01	PCC (CDI_N_)
					–12.96	–0.303	<0.01	PCC (CDI_P_)

*The volume, center of mass coordinates in MNI space, regression parameter (β), linear correlation coefficient (*r*), statistical significance (*p*), and anatomic annotations are specified. The default is bilateral, while R and L- indicate the right and left hemisphere of the brain, respectively. CDI_*NP*_ indicates the overlapping results between CDI_*N*_ and CDI_*P*_.*

**FIGURE 7:**
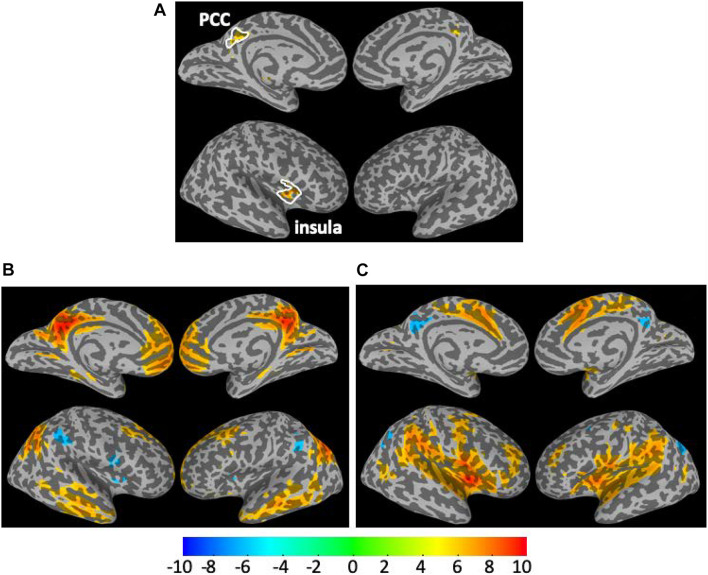
The overlapping ROIs in the PCC and right insula cortex where both the CSI_P_ and CSI_N_ metrics depict significant decline with the adult age **(A)**. The one sample *t*-test maps for the Pearson’s correlation maps associated the seeds defined as the overlapping ROI in the PCC **(B)** and insula cortex **(C)**.

(1) The CSI_P_ shows mainly decline trend with adult age (negative β and *r*) in the extended DMN including superior and MFG, PCC, bilateral insula cortex and left middle temporal gyrus (l-MTG) except for putamen where upregulation of CSI_P_ was observed.
(2) The CSI_N_ depicts a more complicated pattern of dependence on adult age. The negative connectivity strength was reduced (positive β and *r*) with adult age in the PCC, right insula cortex and IPL, while enhancement (negative β and *r*) was detected in the sensorimotor network (paracentral lobule, bilateral postcentral gyri), bilateral parahippocampal cortices (PHC), and right superior temporal gyrus.
(3) There are two brain regions where both the CSI_N_ and CSI_P_ demonstrated significant reduction trend with adult age, which were detected by applying the logical “AND” operation to the regression results for the CSI_P_ and CSI_N_. As shown in Table 2 and Figure 7, the two overlapping ROIs in the PCC and r-insula cortex depict significant down-regulation of CSI_P_ and CSI_N_ metrics with the subjects’ age.

**FIGURE 8:**
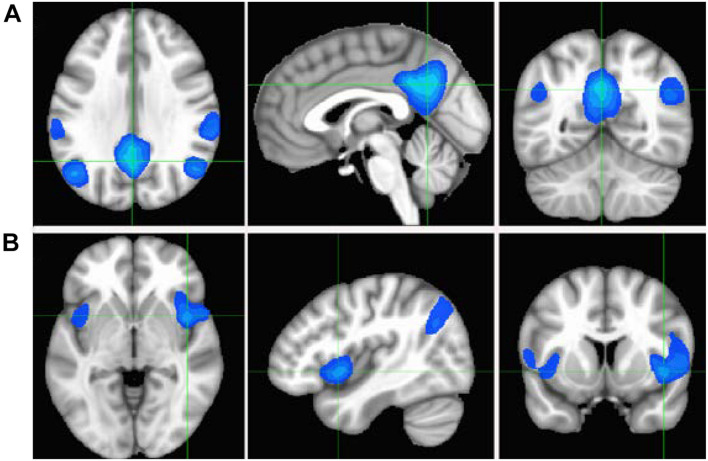
Cross-sectional display of the anti-correlation network associated with the PCC **(A)** and insula **(B)** seeds as derived by multiplying the correlation maps shown in Figures 7B,C and applying a negative threshold at CC ≤ (−0.5). The crossing points of green lines depict the center of mass for PCC and insula ROIs.

To study the specific connectivity associated with the two ROIs defined by the overlap between the CSI_N_ and CSI_P_ metrics, we computed Pearson’s correlation maps for the time courses of the seeds as defined by the overlapping ROIs depicted in Figure 7A. As expected, the associated RFC network for the PCC ROI is obviously the well-known DMN and include 4 negatively correlated brain regions, which are the bilateral IPL and insula cortices (see Figure 7B). On the other hand, the associated RFC network for the insula ROI includes the PCC and bilateral precuneus as the negatively correlated brain regions (Figure 7C) Figure 8. Shows the anti-correlated brain regions between the above 2 RFC networks as obtained by multiplying the two correlation maps with each other and applying negative threshold at CC ≤ (−0.5). The mutually inclusive anti-correlation between the PCC and the right insular cortex explains why both CSI_P_ and CSI_N_ metrics in these regions depict declines with the adult age.

**FIGURE 9:**
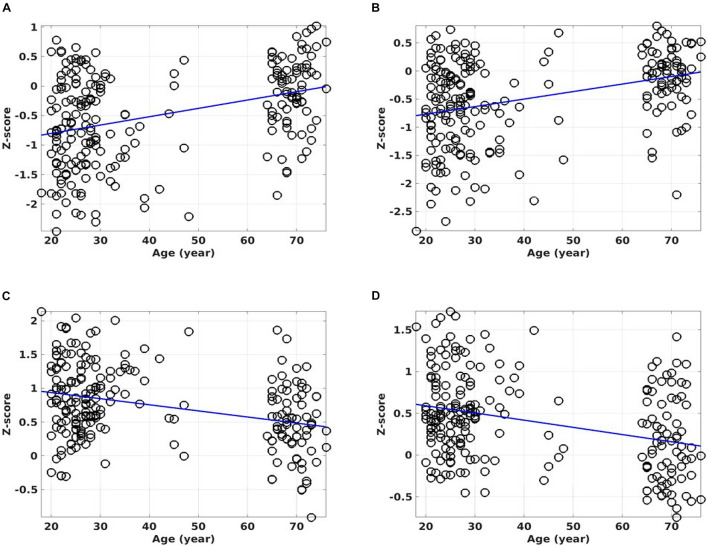
Scattered plots of the regression against age for the overlapping results between the CSI_N_ and the CSI_P_ metrics (see the bottom rows of Table 1). The ROI average of the CSI_N_ metric against the subject’s age for the overlapping ROI in the PCC **(A)**. The ROI average for the CSI_N_ metric against the subject’s age for the overlapping ROI in the right insula cortex **(B)**. The ROI average for the CSI_P_ metric against the subject’s age for the overlapping ROI in the PCC **(C)**. The ROI average for the CSI_P_ metric against the subject’s age for the overlapping ROI in the insula cortex **(D)**. The lines show the linear regression results of the RFC metrics against the subject’s ages.

Figure 9 shows the ROI average of the CSI_N_ and CSI_P_ metrics in the PCC and right insula cortex as a function of the subject’s age. With normal aging, both the CSI_P_ and CSI_N_ are reduced in these brain regions (overlap shown in Figure 7A). Therefore, the PCC and right insula are particularly sensitive to the adult age effect. However, the aging effect is barely detectable by the unseparated CSI metric (see Table 1).

**FIGURE 10:**
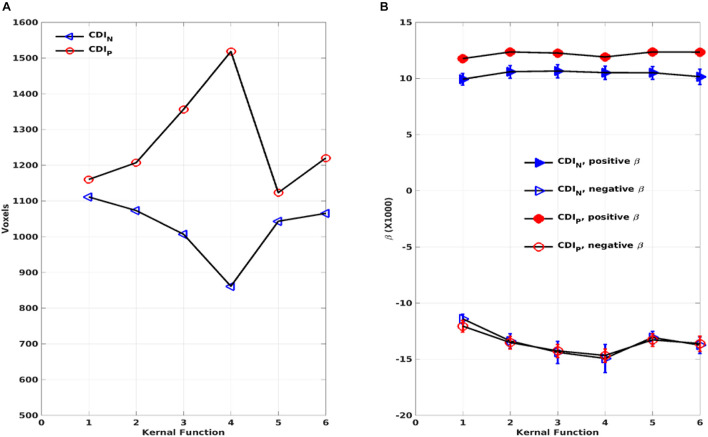
The total volumes of the detected brain regions with significant correlation (*p* < 0.05, corrected) between the connectivity density index (CDI_P_ and CDI_N_) and the subject’s age as a function of the kernels **(A)**. The average regression parameter β for the detected brain regions as a function of the kernels **(B)**. The negative and positive correlations were assessed separately.

As expected, the CDI_P_ and CDI_N_ metrics derived by using the different kernels differ in their sensitivity in detecting the adult age effect. Figure 10A shows the detected brain volumes where the CDI_P_ and CDI_N_ metrics are significantly associated with the adult age. The sensitivity difference of the kernels is also manifested in the regression parameter β which are detailed in Table 3 and Figure 10B. To compare the similarity of the detected aging effects among the CDI metrics of different kernels, we assessed the joint overlapping brain regions detected by the different CDI_N_ and CDI_P_ metrics of different kernels. The observed overall trends of RFC enhancement or decline with age are quite similar. The joint overlapping volumes for the CDI_P_ and CDI_N_ metrics of different kernels are 733 and 671 voxels, respectively. Moreover, there is also a reasonable anatomic consistency between the results of the connectivity strength metrics and connectivity density metrics. As detailed in Tables 2, 3, the anatomical locations of the joint overlapping regions for the different CDI_P_ metrics match those for the 3 largest ROIs identified by the CSI_P_ results (see Table 1). Similarly, the brain regions of the joint overlapping for the different CDI_N_ metrics are largely the same as those identified by the CSI_N_ data (see Table 1). However, it should be noted that the β parameters for the CDI_N_ and CSI_N_ have opposite signs even through the trend of change with the adult age is the same. This is because the negative connectivity strength (CSI_N_) is negative in nature, while the connectivity density corresponding to the negative correlation (CDI_N_) is always positive. Therefore, the enhancement of the negative connectivity strength (CSI_N_) with age (for example in the sensorimotor network) corresponds to a negative β, while the connectivity density result corresponds to a positive β value.

**TABLE 3:** The joint overlapping brain regions where the connectivity density metrics of different kernels are all significantly (*p* < 0.05) correlated with the subjects’ ages.

RFC	Voxel	*X*_cm_	*Y*_cm_	*Z*_cm_	β (10^3^)	*r*	*p*(10^–3^)	Notations
CDI_N_	216	+ 0.1	+ 51.9	+ 29.2	–13.81	–0.376	<0.01	PCC
	103	+ 0.5	+ 28.2	+ 57.6	12.16	0.366	<0.01	Paracentral lobule
	92	–48.2	+ 47.0	+ 42.1	–13.32	–0.431	<0.01	R-IPL
	72	–38.7	+ 26.2	+ 57.2	11.91	0.326	<0.01	R-post central gyrus
	44	+ 21.2	–4.7	–20.6	9.95	0.430	<0.01	L-PHC
	38	–41.9	–12.4	+ 5.0	–15.16	–0.374	<0.01	R-insula cortex
	29	+ 20.8	–4.0	–20.0	8.75	0.371	<0.01	L-STG
	29	–1.5	–14.4	+ 39.5	–11.66	–0.355	<0.01	Anterior cingulate cortex
	28	+ 44.0	+ 24.1	+ 57.1	10.37	0.297	<0.01	L-post central gyrus
	20	–45.2	+ 18.4	+ 9.6	9.20	0.364	<0.01	R-STG
CDI_P_	567	+ 4.2	–46.9	+ 24.8	–12.20	–0.480	<0.01	Superior and MFG
	136	–9.4	+ 21.2	–22.3	8.53	0.452	<0.01	Putamen
	30	+ 57.0	+ 15.8	–15.8	–10.09	–0.387	<0.01	L-MTG

*The volume, center of mass coordinates in MNI space, and anatomic annotations, regression parameters (β), linear correlation coefficient (*r*), statistical significance (*p*), and anatomic annotations are specified. The default is bilateral, while R and L- indicate the right and left hemisphere of the brain, respectively. CDI_*N*_ and CDI_*P*_ indicate the joint overlaps among the CDI_*N*_ and CDI_*P*_ metrics of the different kernels, respectively. The β, *r*, and *p* are the average results for the 6 different kernels.*

## Discussion

### Effects of Adult Age on Resting-State Functional Connectivity

Age is an important risk factor for declines of neural cognitive functions and pathology of neurodegenerative diseases. It is also a complex metric difficult to precisely interpret the involved physiology. Healthy individuals of similar age may have quite different vascular and brain-health status. It follows that age is not a single strongest predictor for the RFC in the brain. This is likely to be the reason why the linear regressions of the RFC metrics with the adult age depict substantial scatters and relative low correlation coefficients. The impact of the potential confounds and pre-processing strategies that can mitigate them have been extensively investigated in the published literature (Bluhm et al., 2008; Biswal et al., 2010; Weissman-Fogel et al., 2010; Zuo et al., 2010; Dennis and Thompson, 2014; Alarcon et al., 2015; Zhang C. et al., 2016; Geerligs et al., 2017; Hussein et al., 2020). Here we focus on comparing our findings in the context of documented literature results, particularly the adult age effect in the DMN, dorsal attention network (DAN), sensorimotor network, and subcortical brain regions.

**FIGURE 11:**
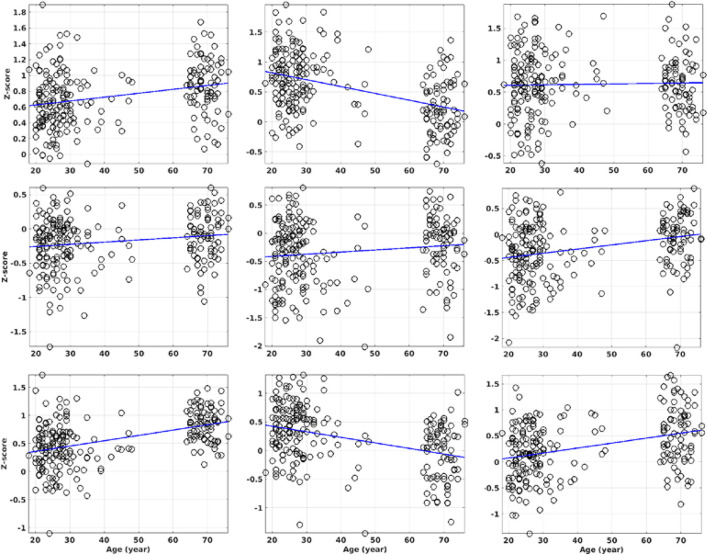
The ROI average of the CSI_P_, CSI_N_, and CSI metrics against the subject’s age for the 3 ROIs with significant correlation between CSI and the subject’s age. The details of the regression results are summarized in Table 4. The columns 1 to 3 are the results for the ROIs in the precuneus, MFG, and R-IPL, respectively. The rows 1 to 3 are the results for the CSI_P_, CSI_N_ and CSI metrics, respectively. The ROI masks are solely based on the CSI metric only.

With QDA, we found support for RFC decline with advancing adult age in multiple brain regions of the DMN and DAN, including superior and MFG, PCC, MTG, and IPL. Age-related RFC decrements in the DMN and DAN have previously been reported in numerous R-fMRI studies using ROI and ICA based analysis (Buckner et al., 2008; Damoiseaux et al., 2008; Dennis and Thompson, 2014; Scheinost et al., 2015; Luo et al., 2020). Our findings regarding to the RFC changes in the DMN are overall in agreement with previous reported results (Damoiseaux et al., 2008, 2016; Koch et al., 2010; Ystad et al., 2010; Williams, 2013; Lu et al., 2014; Persson et al., 2014, 2015; Salami et al., 2014). Besides the DMN and DAN, normal aging was associated with RFC increase in the sensorimotor, subcortical network, and para-hippocampal cortex. This has also been reported previously (Persson et al., 2015, 2016; Damoiseaux et al., 2016; Geerligs et al., 2017; Hussein et al., 2020; Luo et al., 2020). We didn’t find significant age-related RFC declines in precuneus and specific sub-regions of the hippocampal cortex as reported in previous studies (Salami et al., 2014; Damoiseaux et al., 2016). Since we assessed the negative and positive correlation separately, this may allow us to detect more intricate age-related RFC changes in the brain. To illustrate this point, we analyzed further the 3 ROIs with significant correlation between the CSI and the subject’s age. As shown in Tables 1, 4 and Figure 11, the detected ROI in the precuneus depicted significant positive linear correlation between CSI and the subject’s age (β = 9.50 × 10^–3^, *r* = 0.459), even though the CSI_P_ and CSI_N_ in the same ROI showed only a slight (not significant) increment and decrement with age, respectively, i.e., contribution from a low-significant CSI_P_ increment and a non-significant CSI_N_ decrement resulted in a highly significant increment trend in the CSI metric. With the same line of reasoning, we can explain why the MFG ROI detected by the CSI metric is much smaller than that detected by the CSI_P_ metric, because the decremental trend in the CSI_P_ metric was partially canceled by the CSI_N_ contribution. This can also explain why we didn’t detect significant CSI decrement with the adult age in the PCC and R-insula, because both the CSI_P_ and CSI_N_ metrics exhibited significant decremental trends with age and their contributions annulled each other. Therefore, it is important to pay attention to the precise definition of the RFC when comparing the results of different studies.

**TABLE 4:** The linear regression results for the 3 ROIs with significant correlation between CSI and the subject’s age.

ROI	CSI_P_			CSI_N_			CSI		
			
	*β × 10^3^*	*r*	*p*	*β × 10^3^*	*r*	*p*	*β × 10^3^*	*r*	*p*
Precuneus	4.93	0.283	<0.01	3.17	0.175	0.07	9.50	0.459	<0.01
MFG	–11.46	–0.441	<0.01	3.77	0.136	0.11	–9.72	–0.389	<0.01
R-IPL	0.73	0.031	0.61	8.03	0.299	<0.01	9.57	0.354	<0.01

*The CSI_*P*_ and CSI_*N*_ results are based on the masks determined solely by the CSI results.*

Both CSI_P_ (*r* = 0.506, see Table 1) and CDI_P_ (*r* = 0.577, see Table 2) showed age-related enhancement Caudate/putamen and the association are quite strong. This finding based on QDA approach are consistent with previous reports (Manza et al., 2015; Rieckmann et al., 2018) from ROI-based studies aimed to investigate the aging effect on specific functional connectivity of in the striatum-cortical system. It is well known that the striatum-somatomotor connection is primarily associated with motor performance, especially the “automatic” performance of already learned movements. It has been reported that posterior putamen and pallidum decreases in connectivity to left somatomotor cortex with age (Manza et al., 2015). This provides a reasonable explanation for the commonly observed motor deficits as in elderly subjects. There is also growing evidence support the notion that striatum-cortical connectivity is potentially important for memory function at older age. Intriguingly, this is related to the enhanced RFC with age in the putamen/caudate. Several studies have reported that increased striatum functional connectivity in older adults typically reflects less negative connectivity between two regions belonging to different networks, and the increased connectivity is often negatively associated with cognitive performance (Rieckmann et al., 2018). Therefore, better understanding the RFC change with adult age in the striatum-cortical system can be potentially useful for assessing motor as well cognitive functions in elderly subjects.

### Methodological Issues

The QDA framework proposed in the study is a voxel-wise and data-driven approach. It has the following two unique features: (1) It can avoid confounding caused by the cancelation of the negative and positive correlations by assessing the negative and positive portions of the CC histogram separately; (2) It derives different RFC metrics based on the connectivity strength and density by utilizing the concept of convolutions with different kernels. The metrics weight all the correlations of a given voxel with the rest of the brain according to the amplitudes of the correlation coefficients and disregard the anatomical distance between the correlation pairs. This permits a comprehensive characterization of the intrinsic activities of each voxel without the use of an arbitrary threshold. The QDA approach can encapsulate the widely used threshold approach as a special case of the square-well kernel function. The widely used degree centrality corresponds precisely this square-well kernel situation which adopts a somewhat arbitrary threshold and every connection above the threshold are weighted equally. Even the CSI metrics can be encapsulated under the convolution concept for a special kernel of the sign function. This provides a unified view for RFC and can facilitate its further optimization. The QDA framework uses the time course of each voxel within the brain as the seed reference to compute voxel-wise whole-brain correlational coefficient matrix. For whole-brain R-fMRI data acquired at 4 mm spatial resolution, the correlational coefficient matrix is in the order of 10^5^ and is currently not practical for direct visualization and statistic assessment. Particularly, when data are acquired with higher spatial resolution, e.g., 2 mm, the matrix size is increased by 8 × 8 times. Therefore, for data reduction, we derived two types of voxel-wise RFC metrics from the correlational coefficient matrix without the need for specifying a particular seed, threshold, model, or mode. As their names indicated, CSI and CDI are aimed to capture the local (voxel) connectivity strength and density with rest of brain, respectively. The QDA metrics can assess the general connectivity with the rest of the brain without specifying a specific path or network. The QDA method does not highlight the specific connectivity changes between selected brain regions. The precise neural correlate of R-fMRI signal is currently not well understood (Hyder and Rothman, 2010). However, it is reasonable to assume that the R-fMRI signal fluctuations indirectly reflect the slow modulations of neuronal activities at rest. Furthermore, the sigmoid function has been widely accepted as the logistic function of neuronal activation instead of a square-well. With current status of knowledge, we cannot identify a convolution kernel to reveal a particular feature of the neurophysiology. However, we can attempt to optimize the kernel to reduce bias and improve sensitivity of the RFC metrics to pathophysiological changes.

The current results based on the QDA framework should be interpreted in the context of some technical and biological limitations. Firstly, at a TR of 2,500 ms, the cardiac and respiratory fluctuation effects might be aliased into the low frequency R-fMR signal fluctuations. The regression up-to the 1st order derivative of the head motions and lowpass filtering could not eliminate the effects of these physiological noises (Muschelli et al., 2014; Pruim et al., 2015; Bright et al., 2017; Parkes et al., 2018). Thus, these aliasing effects could reduce the specificity of the RFC metrics, or even might further confound the detected RFC differences between the young and elderly sub-groups. With the more up-to-date acquisition techniques, such as multi-band simultaneous acquisition of multiple slices and compress-sensing with high under-sampling factor, it is possible to use a shorter TR (e.g., 500 ms) and higher spatial resolution for the data acquisition. Therefore, these physiological effects may be further mitigated.

Secondly, the resting state is associated with spontaneous thoughts and cognitive processing, we cannot exclude the possibility that differences in spontaneous thoughts may exist between the young and elderly subjects (Wu et al., 2007). However, considering the overall consistency of our results with the previous studies, particularly the results from the longitudinal studies (Fjell et al., 2014; Ng et al., 2016; Staffaroni et al., 2018; Li Q. et al., 2020), it is unlikely that these differences have major influence on our findings. These initial findings encourage the future use of QDA as a tool to analyze longitudinal R-fMRI data aimed to develop a comprehensive understanding of age- or pathology-related brain functional changes.

Thirdly, the generalizability, or external validity issue should be considered. This is due to the non-random recruitment procedures and relying on a sample of convenience. The sample size used in this study (*N* = 227) is moderate, includes unbalanced young and elderly subgroups reflecting the difficulties to recruit elderly healthy subjects. The ages of the participants range from young to old adulthood (reflecting the age of participants in most neuroimaging studies). The age-related RFC differences observed in this study were relatively small but quite robust. However, the results from this cross-sectional study of the cohort cannot distinguish whether the RFC changes in the brain regions are due to gradual changes throughout the adulthood or a more sudden change at later stage in life.

Fourthly, the R-fMRI data were acquired under open-eye condition. Recent studies indicate that opening versus closing eyes at resting-state results in RFC difference between V1 with DMN and SNs (Costumero et al., 2020). This may explain why we did not detect significant RFC change with age in the visual cortex.

### Negative Cross Correlation in White Matter and Cerebral Spinal Fluid

As discussed above negative correlation is an important fraction of the CC histogram irrespective of the tissue type and anatomical location of the voxel in question. In the published literature, there is also a rapid growing interest in studying the negative correlations between the voxels (Fox et al., 2009; Weissenbacher et al., 2009; Bianciardi et al., 2011; Schwarz and McGonigle, 2011; Gonzalez-Castillo et al., 2012; Gopinath et al., 2015; Liu et al., 2015; Spreng et al., 2016; Chen et al., 2020). It is clear that the negative portion of the CC histogram is more dominant for voxels in CSF (Gruszecki et al., 2018) and white matter (McColgan et al., 2017; Gore et al., 2019; Wu et al., 2019; Li M. et al., 2020). However, the negative portion cannot be ignored even for voxels in the gray matter (see Supplementary Materials). To avoid confound caused by inappropriate preprocessing pipelines, we have carefully tested and updated our preprocessing pipeline. We did not implement the global signal regression (GSR) which removes the mean signal averaged over the entire brain. GSR removal via linear regression is one of the most controversial procedures in the analysis of R-fMRI data (Fox et al., 2009; Weissenbacher et al., 2009). On one hand, the global mean signal contains variance associated with respiratory, scanner-, and motion-related artifacts. Its removal by GSR can improve various quality control metrics, which enhances the anatomical specificity of RFC networks, and increase the explained behavioral variance. On the other hand, GSR alters the distribution of regional signal correlations in the brain, can induce artefactual anti-correlation patterns, may remove real neural signal, and can distort RFC metrics. The brain masked “global signal” is usually misunderstood, because it is not “global” and its variance contains dominant contributions from different domains of the voxels with temporally coherent signal variation.

To limit the study in a reasonable scope, in the discussion of the adult age effect on RFC we focused on gray matter and did not discuss white matter and CSF related issues. However, it should be pointed out that aging effects in white matter (McColgan et al., 2017; Gore et al., 2019; Wu et al., 2019; Li M. et al., 2020) and CSF (Sakka et al., 2011; Gruszecki et al., 2018) are also worth exploring. There is indeed a rapid growing interest in these arenas in published literature (Sakka et al., 2011; McColgan et al., 2017; Gruszecki et al., 2018; Gore et al., 2019; Wu et al., 2019; Li M. et al., 2020), particularly in the context of the age effect for the glymphatic system.

## Conclusion

The proposed QDA framework can provide data-driven, threshold-free and voxel-wise analysis of R-fMRI data and offer a unified view for RFC metrics which can facilitate further development and optimization of the RFC metrics by choosing appropriate kernel functions. The QDA results for the adult age effect are largely consistent with previously published results based on other analysis methods. Moreover, our new findings based on the separate assessment of the negative and positive correlations can improve the sensitivity of the RFC metrics to physiological changes associated with the advancing adult age and may clarify some of the confounding reports in the literature regarding to the DMN and sensorimotor network involvement in normal aging.

## Data Availability Statement

The datasets presented in this study can be found in online repositories. The names of the repository/repositories and accession number(s) can be found below: http://dx.doi.org/10.17 632/pt9d2rdv46.1.

## Ethics Statement

The studies involving human participants were reviewed and approved by the regional Ethics Committee in Stockholm and the study protocols included 2012/1511-31/2 and 2014/1982-31/1. The patients/participants provided their written informed consent to participate in this study.

## Author Contributions

XL: conceptualization and software. HF: project administration, editing, supervision, and funding acquisition. AM: validation and visualization. KM: investigation and data curation. TQL: methodology, formal analysis, and original draft preparation. All authors contributed to the article and approved the submitted version.

## Conflict of Interest

The authors declare that the research was conducted in the absence of any commercial or financial relationships that could be construed as a potential conflict of interest.

## Publisher’s Note

All claims expressed in this article are solely those of the authors and do not necessarily represent those of their affiliated organizations, or those of the publisher, the editors and the reviewers. Any product that may be evaluated in this article, or claim that may be made by its manufacturer, is not guaranteed or endorsed by the publisher.

## Footnotes

1
http://www.fmrib.ox.ac.uk/fsl

## Supplementary Material

Supplementary Figure 1 Anatomic locations for 4 different seed voxels of different tissue types including white matter (WM), cerebral spinal fluid (CSF), PCC, and motor cortex (MC). The crossing green lines depict the locations of the cross sections.

Supplementary Figure 2 The average CC histograms of the cohort for the 4 different seed voxels shown in Supplementary Figure 1 and Supplementary Table 1.

Supplementary Figure 3 The convolutions of the CC histograms shown in Supplementary Figure 2 for the 4 different seed voxels located in WM (a), PCC (b), CSF (c), and MC (d).

Supplementary Table 1 The MNI coordinates for the 4 voxels of different tissue types illustrated in Supplementary Figure 1.
