# Supplementary material for: A Quantitative Data-Driven Analysis Framework for Resting-State Functional Magnetic Resonance Imaging: A Study of the Impact of Adult Age
Source: Front Neurosci. 2021 Oct 20;15:768418. doi: 10.3389/fnins.2021.768418 (PMC8565286; doi:10.3389/fnins.2021.768418)
Supplement: Supplementary Figure 1 — Anatomic locations for 4 different seed voxels of different tissue types including white matter (WM), cerebral spinal fluid (CSF), PCC, and motor cortex (MC). The crossing green lines depict the locations of the cross sections. [file Image_1.pdf]

Supplementary Materials

For further illustration, we selected 4 seed voxels located in different brain regions and tissue types (see **Supplementary Figure 1** and **Supplementary Table 1**) for further examination.

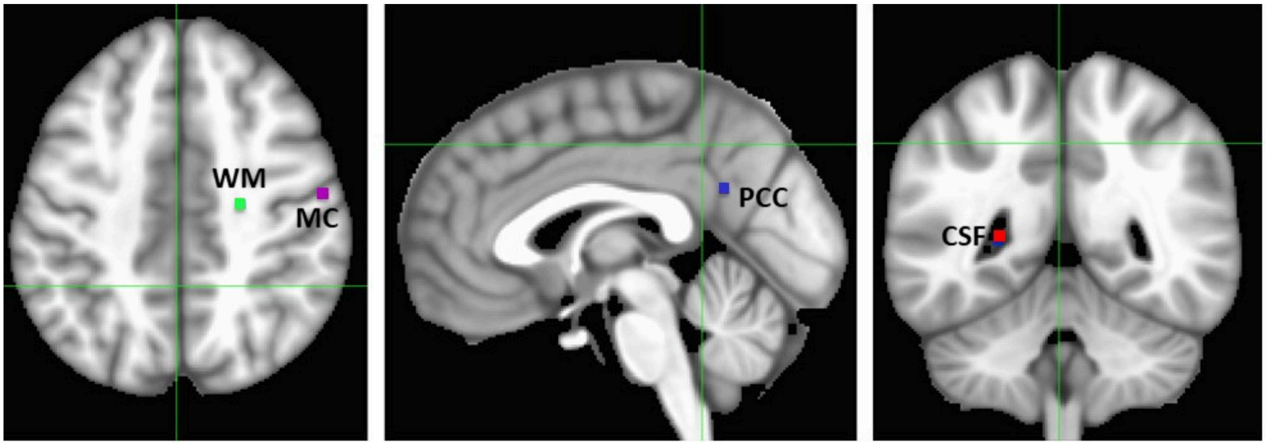

Supplementary Figure 1 | Anatomic locations for 4 different seed voxels of different tissue types including white matter (WM), cerebral spinal fluid (CSF), PCC and motor cortex (MC). The crossing green lines depict the locations of the cross sections.

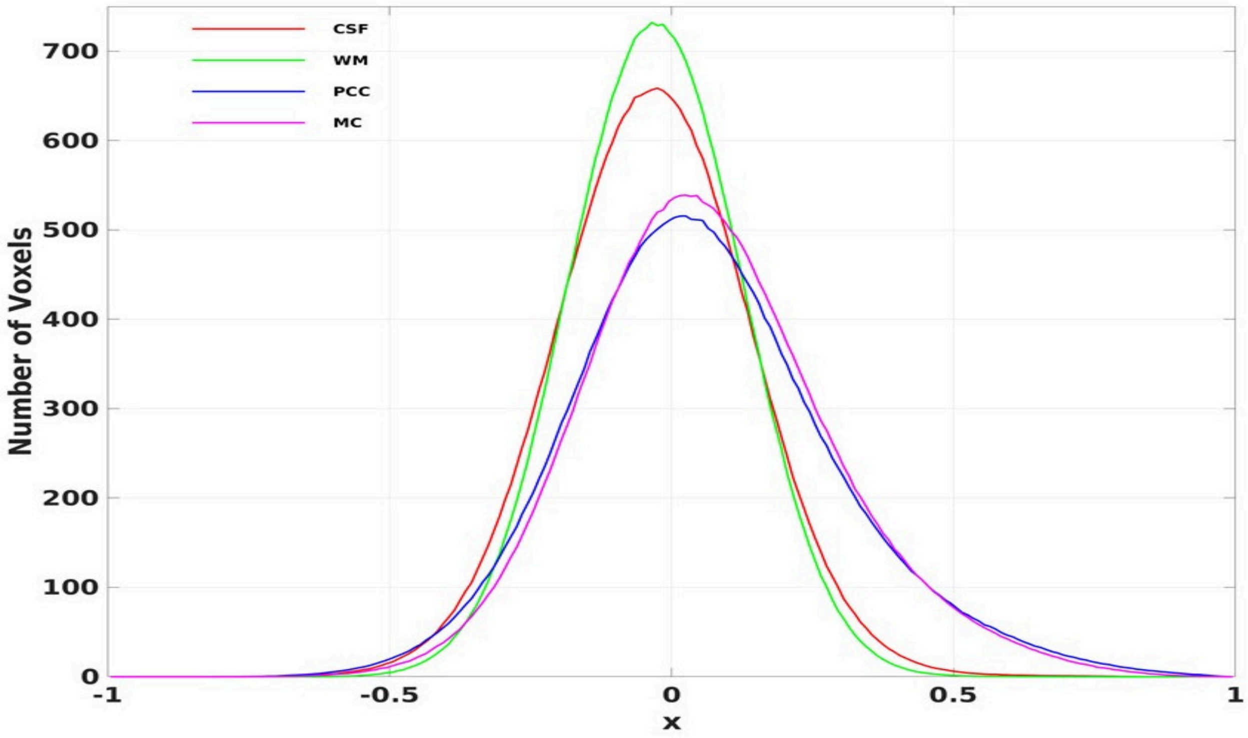

Supplementary Figure 2 | The average CC histograms of the cohort for the 4 different seed voxels shown in **Supplementary Figure 1** and **Supplementary Table 1**.

As shown in **Supplementary Figure 2**, the histogram for the MC seed is quite similar to that for the PCC seed with a long positive tail, whereas the histograms for the WM and CSF seeds are overall narrower and the peak is slightly shifted toward the negative side. The convolutions of these CC histograms are depicted in **Supplementary Figure 3**. For the WM and CSF seeds, the negative portions are more dominant, while the positive parts of the convolutions are larger for the MC and PCC seed voxels in grey matter. For the polynomial kernels, increasing the order of the polynomial shifts the peak values of the convolutions away from "0". Selecting different kernels can, therefore, adjust the contrast and sensitivity of the derived CDI_P and CDI_N metrics.

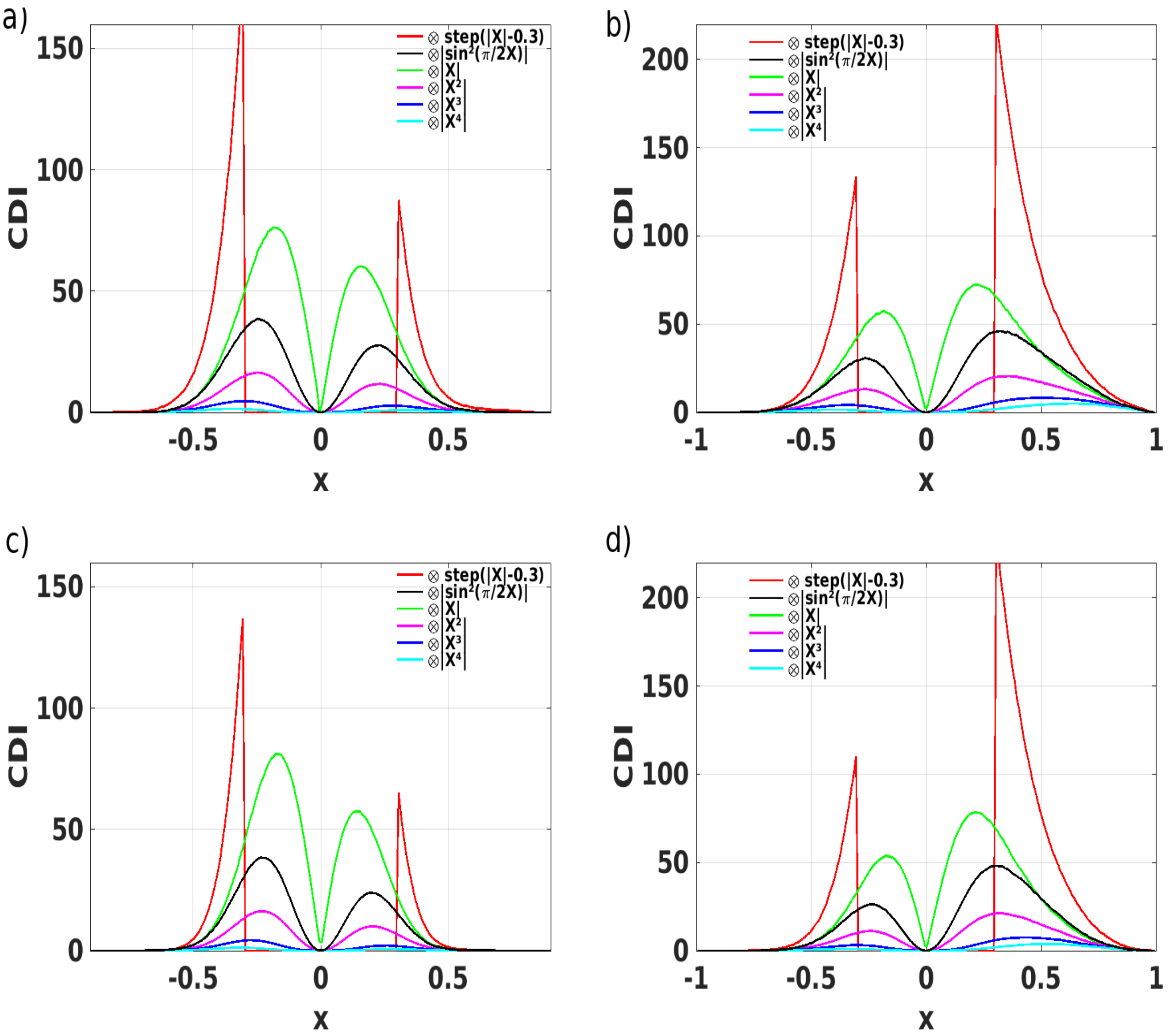

Supplementary Figure 3 | The convolutions of the CC histograms shown in Fig. S2 for the 4 different seed voxels located in WM (a), PCC (b), CSF(c), and MC (d).

Supplementary Table 1 | The MNI coordinates for the 4 voxels of different anatomical locations and tissue types illustrated in **Supplementary Figure 1**.

Tissue	X_{cm}	Y_{cm}	Z_{cm}
CSF	+26	+48	+8
WM	-22	-16	+43
MC	-56	-12	+43
PCC	0	+56	+26
